# Establishment of Laboratory Bioassay System for *Phyllotreta striolata* Larvae and Screening of Novel Bt Cry Proteins

**DOI:** 10.3390/toxins18040191

**Published:** 2026-04-20

**Authors:** Leqi Wang, Zhenyi Liu, Ivan M. Dubovskiy, Changlong Shu, Jie Zhang, Junjie Zhang, Wenmei Du, Qi Peng

**Affiliations:** 1 Engineering Research Center of Natural Enemies, Institute of Biological Control, Jilin Agricultural University, Changchun 130118, China; 2 State Key Laboratory for Biology of Plant Diseases and Insect Pests, Institute of Plant Protection, Chinese Academy of Agricultural Sciences, Beijing 100193, China; 3 Research Center of Biological Plant Protection, Siberian State University of Engineering and Biotechnology, Dobrolubova Str. 160, 630039 Novosibirsk, Russia

**Keywords:** *Phyllotreta striolata*, *Bacillus thuringiensis*, Cry proteins

## Abstract

*Phyllotreta striolata* is a global pest of cruciferous vegetables, and controlling its soil-dwelling larvae is challenging. The lack of standardized larval bioassay methods hinders the screening of effective biocontrol agents. In this study, we established a stable and standardized laboratory-efficacy trial system for *P. striolata* larvae. Indoor rearing techniques were optimized for *Brassica juncea* var. *foliosa* and *Brassica juncea* var. *megarrhiza* were identified as the optimal host plants, with ideal oviposition conditions at 26–28 °C using black flannel substrate, and soil-cultured *Brassica rapa* var. *pekinensis* as the host plant. Based on these findings, a larval bioactivity assay was established using *B. juncea* var. *megarrhiza* slices on water-agar. This system maintained a natural larval mortality rate below 5% within 48 h, meeting the bioassay requirements. The reliability of the system was validated by evaluating the activity of the engineered *Bacillus thuringiensis* (Bt) strain G033A against larvae, where the LC_50_ value decreased from 23.013 mg/mL to 7.295 mg/mL with an extended treatment time (12–48 h). Using this standardized method, novel Cry proteins with high activity against *P. striolata* larvae were screened. Cry8Ca and Cry8Ga proteins exhibited LC_50_ values of 2.243 mg/mL and 1.649 mg/mL, respectively.

## 1. Introduction

The striped flea beetle (*Phyllotreta striolata*) is a globally significant pest of cruciferous vegetables, causing severe economic losses in Asia, Europe, North America, and other regions [1]. *P. striolata* completes its life cycle through four stages: egg, larva, pupa, and adult [2]. Adults feed on leaves, creating numerous small holes, whereas larvae bore into and feed on roots, directly impacting plant growth and marketable yield, often leading to substantial production losses or even complete crop failure [3]. In China, *P. striolata* is recognized as one of the most destructive pests in the production of cruciferous vegetables, such as Chinese cabbage, oilseed rape, and radish [2]. It is prevalent across both northern and southern China, with particularly severe damage occurring in the warmer southern regions owing to multiple generations per year and rapid population growth, resulting in persistently high control pressure.

Current management strategies for *P. striolata* primarily rely on chemical insecticides targeting adults. However, the long-term and extensive use of these chemicals has led to increasing insecticide resistance [4]. Consequently, biological control technologies, which offer diverse modes of action, have emerged as crucial alternative strategies. *Bacillus thuringiensis* (Bt) insecticides are the most widely used microbial pesticides worldwide [5,6]. The genetically engineered strain G033A [7], developed by our laboratory in a previous study, expresses the introduced *cry3A* gene and has been successfully registered and applied for the control of *Phyllotreta striolata*. However, the currently available Bt genes for *P. striolata* control are limited, with only a few families, such as *cry3* and *cry8*, reported to be effective against coleopteran pests [8]. The prolonged and repeated use of a single or few genes poses a high risk of resistance development in the field, necessitating the discovery of novel Bt insecticidal genes with high efficacy against this pest species.

A major challenge in screening novel biocontrol agents is that although Bt insecticidal proteins are most effective against young larvae, the fact that *P. striolata* larvae live in the soil makes them difficult to observe and assay [9]. Adults preferentially feed on a range of cruciferous crops, particularly *Brassica rapa*, *B. juncea*, and *Raphanus sativus*, with feeding preferences influenced by the chemistry of the host plant [10,11]. However, unlike many herbivorous insects that follow the preference–performance hypothesis, oviposition in *P. striolata* is not strictly aligned with the adult feeding preference. Females typically deposit eggs in moist soil near the base of host plants, and larval performance is largely determined by root availability and quality rather than by foliar traits [12,13]. After hatching, larvae feed on roots and develop entirely in the soil, further complicating the direct observation and bioassay of early instars. Therefore, there is an urgent need to establish a standardized and reproducible laboratory-based larval bioassay system. Such a system would allow for the quantitative assessment of key biological control factors, including the lethal concentration (LC50) of Bt Cry toxins, virulence of entomopathogenic fungi (e.g., *Beauveria bassiana*), and growth-inhibitory activity of insecticidal proteins from other microbial sources. Thus, establishing this system would provide a critical technical foundation for the screening and development of environmentally friendly pest management products targeting *P. striolata*.

To address these challenges, we first systematically evaluated the effects of different cruciferous host plants and environmental conditions on adult oviposition and larval performance, and then developed a standardized rearing and bioassay system for *P. striolata*. Using this system, two Cry proteins, Cry8Ca and Cry8Ga, were identified as exhibiting high insecticidal activity against *P. striolata* larvae. The discovery of these novel proteins enriches the arsenal of biological control resources for managing this pest.

## 2. Results

### 2.1. Establishment of Laboratory Population for Phyllotreta striolata

#### 2.1.1. Screening for Optimal Host Plants

**Figure 1:**
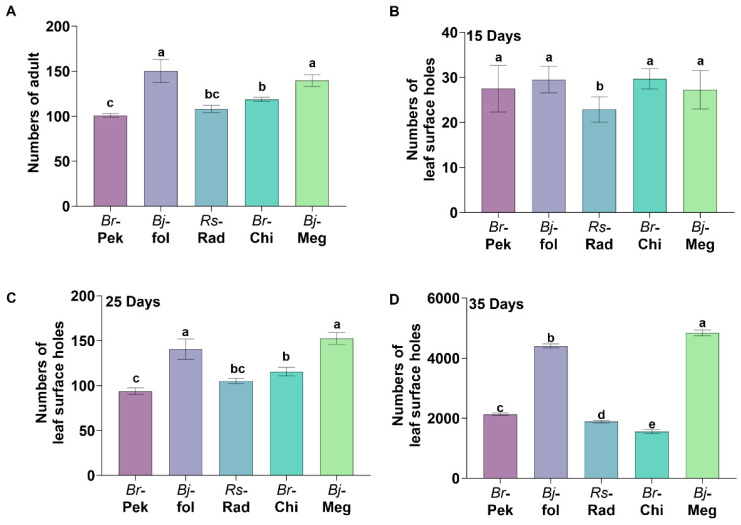
Preference of *Phyllotreta striolata* adults for different host plants. (**A**) Mean daily number of adults captured on each host plant over three days. (**B**–**D**) Mean number of feeding punctures per plant at 15 days (**B**), 25 days (**C**), and 35 days (**D**) after planting. *Br*-Pek: *Brassica rapa* var. *pekinensis*; *Bj*-fol: *Brassica juncea* var. *foliosa*; *Rs*-Rad: *Raphanus sativus* var. *radicula*; *Br*-Chi: *Brassica rapa* var. *chinensis*; *Bj*-Meg: *Brassica juncea* var. *megarrhiza*. Error bars represent SE. Different lowercase letters indicate significant differences among treatments (*p* < 0.001).

To establish a stable laboratory bioassay system for *Phyllotreta striolata* larvae, we optimized indoor rearing techniques based on the field occurrence patterns of the pest, successfully establishing a stable laboratory population. For host plant selection, five candidate species—*Brassica rapa* var. *pekinensis*, *Brassica rapa* var. *chinensis*, *Raphanus sativus* var. *radicula*, *Brassica juncea* var. *foliosa* and *Brassica juncea* var. *megarrhiza*—were evaluated. In experimental plots with a total area of 16 m^2^, the total number of *P. striolata* adults on different host plants was observed daily from 6:00 to 18:00. After three days of observation, the results showed that *B. juncea* var. *foliosa* and *B. juncea* var. *megarrhiza* harbored the highest number of adult beetles, with average daily captures of 150 and 139 individuals, respectively. These were followed by *B. rapa* var. *chinensis* (118 individuals) and *R. sativus* var. *radicula* (108 individuals). *B. rapa* var. *pekinensis* exhibited the lowest adult occurrence, with 101 individuals (Figure 1A).

During the observation period, the number of feeding punctures per plant caused by *P. striolata* was recorded (Figure 1B). At 15 days post-planting, *B. juncea* var. *foliosa* and *B. rapa* var. *chinensis* showed the highest number of feeding punctures per plant, with 29 punctures, respectively, followed by *B. rapa* var. *pekinensis* and *B. juncea* var. *megarrhiza*, both with 27 punctures. *R. sativus* var. *radicula* exhibited the fewest punctures (22 per plant), which was significantly lower than the other four host plants. At 25 d after planting (Figure 1C), *B. juncea* var. *foliosa* and *B. juncea* var. *megarrhiza* had significantly more feeding punctures than the other three hosts, with 152 and 140 punctures per plant, respectively, indicating a feeding preference for these two mustards. At 35 days after planting (Figure 1D), *B. juncea* var. *megarrhiza* exhibited the highest number of feeding punctures (4843 per plant), significantly surpassing all other hosts, followed by *B. juncea* var. *foliosa* (4407), *B. rapa* var. *pekinensis* (2113), *R. sativus* var. *radicula* (1888), and *B. rapa* var. *chinensis* (1556). Significant differences were observed among all five host plants at this time. These results collectively demonstrate that *P. striolata* exhibits a distinct feeding preference for *B. juncea* var. *foliosa* and *B. juncea* var. *megarrhiza.*

#### 2.1.2. Factors Affecting Oviposition of *Phyllotreta striolata* Adults

**Figure 2:**
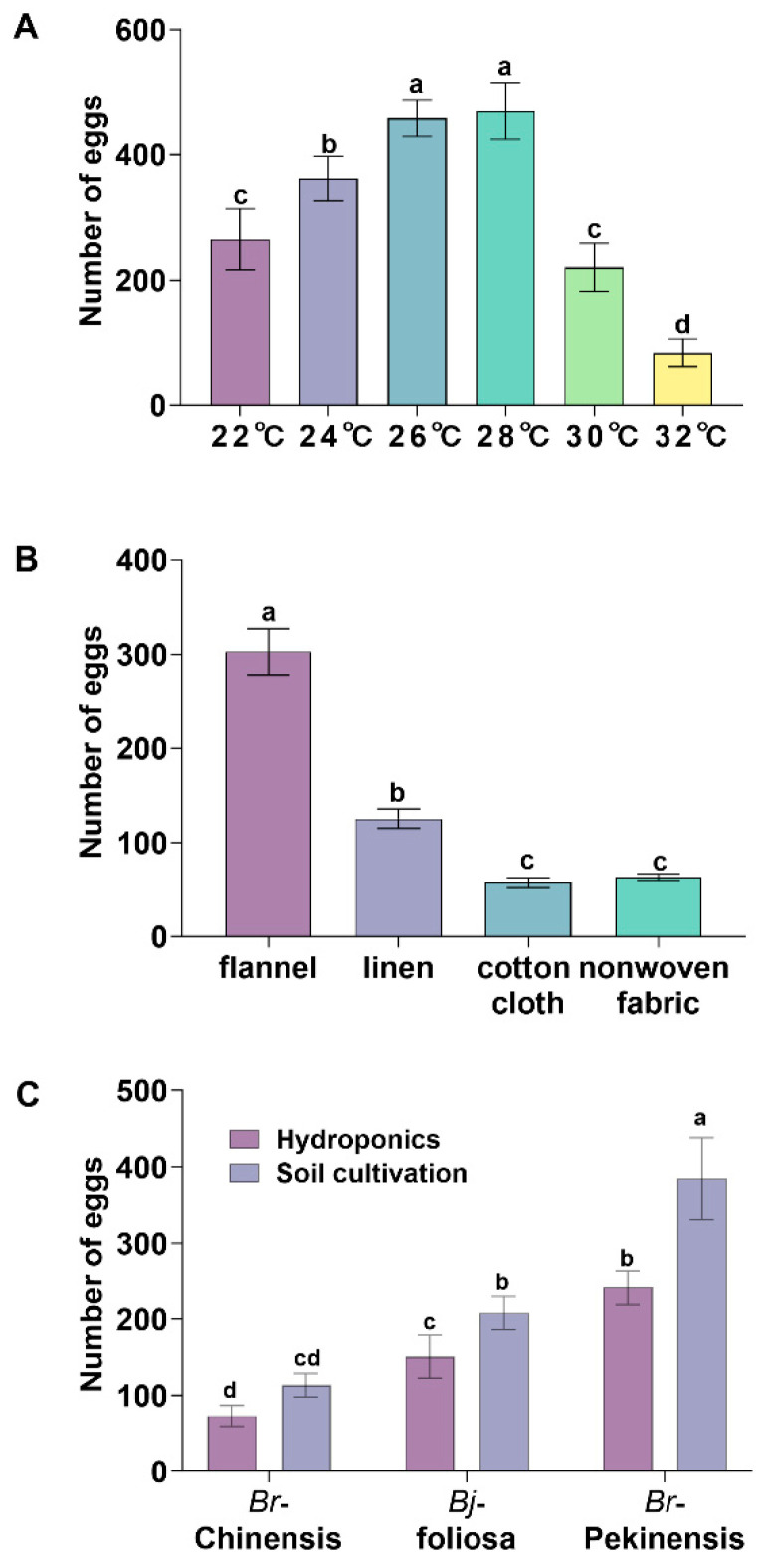
Effects of temperature, oviposition substrate, and host plant on the fecundity of *Phyllotreta striolata* adults. (**A**) Number of eggs laid by 100 adults (male:female = 1:3) under different temperature conditions (22, 24, 26, 28, 30, and 32 °C). (**B**) Number of eggs laid on different oviposition substrates (black flannel, black linen, black cotton cloth, and black nonwoven fabric). (**C**) Number of eggs laid on different host plant roots, soil-cultured vs. hydroponically cultured *B. rapa* var. *pekinensis* (*Br*-pekinensis), *B. juncea* var. *foliosa* (*Bj*-foliosa) and *B. rapa* var. *chinensis* (*Br*-chinensis). Data are presented as mean ± SE. Different lowercase letters above the bars indicate significant differences among treatments (*p* < 0.001).

To optimize larval production, factors influencing adult oviposition were also analyzed. The number of eggs laid by adults under different temperature conditions was also measured. The results showed that *P. striolata* adults laid the highest number of eggs at 26 °C and 28 °C. For 100 adults with a male-to-female ratio of 1:3, the mean number of eggs laid was 457 ± 29 and 469 ± 45, respectively, which were significantly higher than those under other temperature conditions. At 24 °C, the number of eggs laid was 361 ± 34. At 22 and 30 °C, the number of eggs laid was 265 ± 48 and 220 ± 38, respectively. The lowest number of eggs (83 ± 22) was observed at 32 °C (Figure 2A).

The effects of different oviposition substrates on fecundity were further compared. Adults laid the highest number of eggs on black flannel, with an average of 302 ± 24 eggs, which was significantly higher than that on other substrates. Black linen ranked second with 125 ± 10 eggs. Black cotton cloth and black nonwoven fabric yielded the lowest numbers, with 57 ± 5 and 63 ± 3 eggs, respectively (Figure 2B).

Regarding host plants, *P. striolata* laid the highest number of eggs (384 ± 53) on the roots of soil-cultured *B. rapa* var. *pekinensis*, significantly exceeding all other host plant samples (*p* < 0.001). This was followed by hydroponically culturing *B. rapa* var. *pekinensis* roots (240 ± 22 eggs) and soil-cultured *B. juncea* var. *foliosa* roots (207 ± 21 eggs), followed by hydroponically cultured *B. juncea* var. *foliosa* roots (150 ± 28 eggs). The lowest number of eggs was laid on soil-cultured *B. rapa* var. *chinensis* roots (113 ± 15 eggs) and hydroponically cultured *B. rapa* var. *chinensis* roots (73 ± 13 eggs) (Figure 2C). These results demonstrate that the optimal conditions for *P. striolata* adult oviposition are a temperature range of 26–28 °C, soil-cultured *B. rapa* var. *pekinensis* roots as the host, and wrapping with black flannel as the oviposition substrate.

#### 2.1.3. Egg Hatching and Larval Rearing of *Phyllotreta striolata*

**Figure 3:**
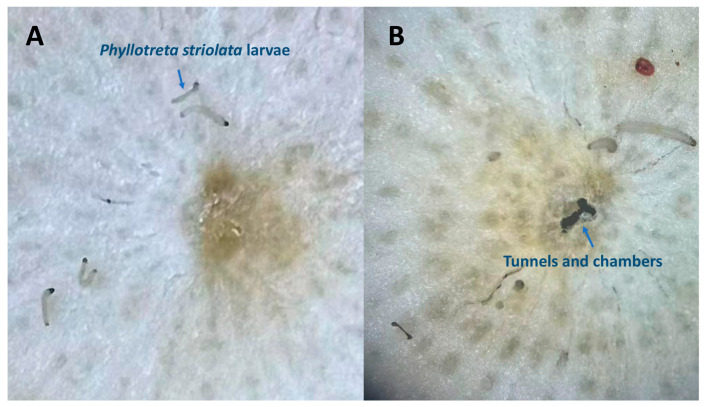
Larval rearing and survival of *Phyllotreta striolata*. Tunnels and chambers excavated by neonate larvae in slices of plump roots of *Brassica juncea* var. *megarrhiza* shortly after hatching. (**A**) 0 h after pest infestation; (**B**) 48 h after pest infestation.

Following adult oviposition, *Brassica juncea* var. *megarrhiza* was selected as the host plant for larval development. Under conditions of 28 °C and 100% humidity, the eggs hatched over a 120 h incubation period, with a hatching rate of 98.3%. Observations revealed that upon hatching, the neonates burrowed into the cross-sections of *B. juncea* var. *megarrhiza*, excavating tunnels and chambers within the root tissue (Figure 3). This demonstrates that *P. striolata* larvae can feed and develop normally under this rearing method, meeting the requirements for bioactivity assays and being suitable for such experiments.

Under the conditions using *B. juncea* var. *megarrhiza* slices of plump roots as feed and water agar to maintain ambient humidity. *P. striolata* larvae grew normally when cultured at 28 °C. The survival rate was 100% for the first 24 h. Minimal mortality began to occur at 36 h, with a survival rate of 98.75 ± 0.02%. At 48 h, the survival rate remained high at 95.8 ± 0.42%. The mortality rate at 48 h of rearing was still low, conforming to the mortality criteria required for bioactivity assays, thereby confirming its suitability for such determinations.

### 2.2. Establishment of a Larval Bioassay System for Phyllotreta striolata

**Figure 4:**
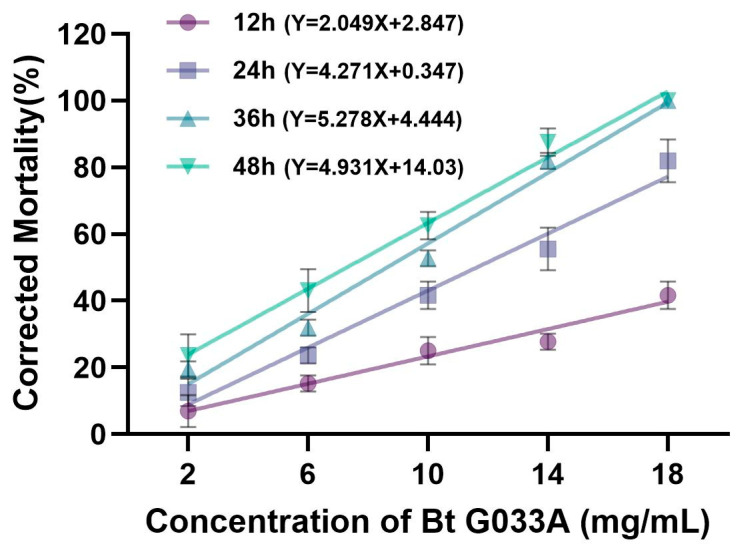
Efficacy of Bt G033A against *Phyllotreta striolata* larvae. Corrected mortality rates of larvae treated with different concentrations of G033A bacterial suspension (2–18 mg/mL) at various time points (12–48 h). Sterile water was used as the control. Data are presented as mean ± SE.

Based on the above findings, a larval bioassay method was evaluated using the Bt product G033A (registration no. PD20171726) and *Brassica juncea* var. *megarrhiza* as the feed. The engineered *Bt* strain G033A powder (32,000 IU/mg) was diluted with water to prepare five concentrations: 2, 6, 10, 14, and 18 mg/mL. *B. juncea* var. *megarrhiza* slices (5 mm thick, 3 cm in diameter) were immersed in bacterial suspensions of each concentration, while discs immersed in sterile water served as the control. The results (Figure 4) showed that after treatment with the G033A bacterial suspension, the corrected mortality of *P. striolata* larvae was positively correlated with both the treatment duration and concentration. Specifically, longer treatment times and higher concentrations resulted in increased corrected mortality rates. At 36 h post-treatment, the corrected mortality reached a maximum of 100% at 18 mg/mL. Based on the bioassay results, regression analysis was performed on the treatment concentrations and larval mortality. The LC_50_ values of the G033A bacterial suspension against *P. striolata* larvae at 12, 24, 36, and 48 h post-treatment were 23.013 mg/mL, 11.626 mg/mL, 8.631 mg/mL, and 7.295 mg/mL, respectively. The LC_50_ values decreased progressively with an increase in exposure time. These results demonstrate that the larval bioassay system established in this study is stable and reliable, making it suitable for screening bacterial strains or proteins with insecticidal activity against *P. striolata*.

### 2.3. Screening of Cry Proteins with High Insecticidal Activity Against Phyllotreta striolata Larvae

**Figure 5:**
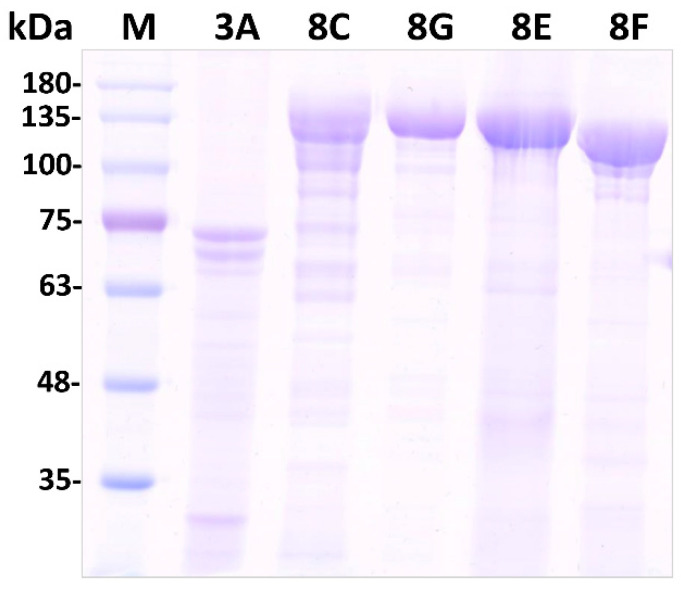
SDS-PAGE analysis of purified Cry proteins. M: Protein molecular weight marker. Lanes 1–6: Marker, Cry3A, Cry8C, Cry8G, Cry8E, and Cry8F, respectively. Each protein migrated as a single band, indicating its high purity.

Using the established bioassay method described above, we screened for insecticidal proteins that were highly effective against *P. striolata* larvae. Cry8 family proteins, including Cry8Ca, Cry8Ga, Cry8Ea, and Cry8Fa, were extracted from Bt strains using the alkaline lysis method established in our laboratory. Cry3A protein extracted from the HD3A strain was used as a positive control. SDS-PAGE analysis of the extracted Cry proteins revealed that each protein migrated as a single, distinct band (Figure 5), indicating their high purity. The extracted Cry proteins were quantified using BSA as a standard and ImageJ software (version 1.54p ), enabling their subsequent use in insecticidal activity assays.

**Figure 6:**
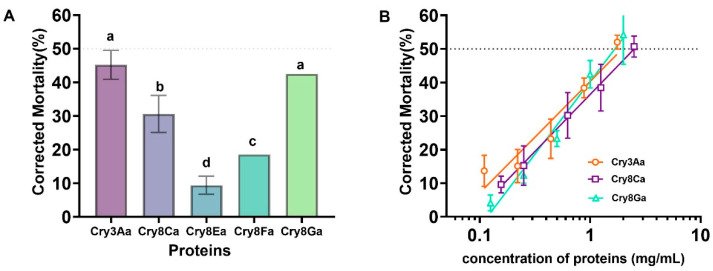
Insecticidal activity of Cry proteins against *Phyllotreta striolata* larvae. (**A**) Corrected mortality of second-instar larvae treated with different Cry proteins at 1 mg/mL after 48 h of treatment. PBS buffer was used as a negative control. (**B**) LC_50_ values of Cry8Ca and Cry8Ga proteins against second-instar larvae after 48 h of exposure. Data are presented as mean ± SE. Bars annotated with the same letter do not differ significantly from each other (*p* < 0.001), whereas bars annotated with different letters indicate statistically significant differences.

The extracted insecticidal proteins were diluted to 1 mg/mL (containing 0.15% Tween-20) for the preliminary screening. The bioassay results (Figure 6A) showed that Cry3A, Cry8Ca, and Cry8Ga proteins exhibited corrected mortality rates greater than 30% against *P. striolata* larvae, whereas the corrected mortality rate for Cry8Ea was less than 10%. Based on the preliminary screening results, proteins with higher insecticidal activity were selected for median lethal concentration (LC_50_) determination. Five concentration gradients were set for each protein, with three replicates for each concentration. Corrected mortality was recorded after 48 h of incubation, and the LC_50_ values were calculated. The results (Figure 6B) showed that the LC_50_ values of Cry8Ca and Cry8Ga proteins against *P. striolata* larvae were 2.243 mg/mL and 1.649 mg/mL, respectively, indicating that both Cry8Ca and Cry8Ga proteins exhibited high insecticidal activity against *P. striolata* larvae.

## 3. Discussion

*Phyllotreta striolata*, a global pest of cruciferous vegetables, poses significant challenges for chemical control because of the cryptic feeding behavior of its larvae within the soil [10,14]. For an extended period, pest management has relied heavily on chemical insecticides, which has not only led to prominent problems of insecticide resistance but has also raised concerns regarding pesticide residues and environmental contamination [13]. *Bacillus thuringiensis* (Bt), an environmentally friendly biological control agent, represents an important approach to address these challenges. However, the Bt products currently registered for the control of coleopteran pests, including *P. striolata*, are limited in diversity [8]. To date, the genetically engineered strain G033A, which expresses the *cry3A* gene, is the only registered Bt product in China specifically developed for the control of *P. striolata* and other coleopteran pests. The prolonged use of insecticidal proteins with a single mode of action increases the risk of resistance development in target pests, and there have been reports both domestically and internationally regarding the development of resistance to the Cry3A protein in various pests [15,16,17]. Therefore, the continuous discovery of novel, highly active insecticidal proteins with different mechanisms of action is urgently required to extend the durability of Bt products and achieve sustainable green pest management. This study not only provides a critical technological platform for the efficient screening of such biological control agents but, more importantly, has directly identified two novel candidate protein resources. These findings lay the foundation for the development of next-generation Bt insecticides with multiple modes of action, which are of great significance for managing chemical pesticide resistance and preventing potential future resistance to Bt proteins in the target pests.

A stable and reproducible laboratory bioassay method is fundamental for screening and evaluating the efficacy of biological control agents. However, a standardized bioassay protocol for the soil-dwelling larvae of *Phyllotreta striolata* is currently lacking. Most existing methods are cumbersome to operate and have inconsistent condition controls, often leading to high larval mortality rates and considerable fluctuations in experimental results, which severely restricts the screening efficiency of highly effective strains or insecticidal proteins. For instance, Chen et al. employed a food immersion method in which radish blocks were immersed in fungal suspensions, air-dried, and then inoculated with second-instar larvae. Fresh radish blocks were replaced daily, and mortality was recorded on the 9th day post-treatment to calculate the LC_50_ values [18]. This bioassay method has a relatively long overall cycle and low efficiency, making it difficult to meet the requirements of large-scale screening. Moreover, the frequent replacement of feed and handling of larvae increases the risk of mechanical damage to test insects, leading to elevated mortality rates and unstable assay results. In this study, we established a bioassay system suitable for *P. striolata* larvae by systematically optimizing key experimental conditions. Specifically, we screened and identified *Brassica juncea* var. *megarrhiza* as the optimal host plant for larval feeding; determined the most favorable oviposition conditions (temperature 26–28 °C, soil-cultured *Brassica rapa* var. *pekinensis* roots as the host, wrapped with black flannel); and innovatively adopted a feeding method using *Brassica juncea* var. *megarrhiza* slices combined with water agar for moisture retention. This system significantly reduced the natural mortality rate of larvae (below 5% within 48 h) and offers advantages such as ease of operation and stability.

Furthermore, prolonged feeding on insects can lead to the decay of plant tissues, which adversely affects larval feeding and bioassay accuracy. Replacing the feed requires detaching larvae from the old feed, which can easily cause larval injury and compromise the reliability of the mortality data. The system established in this study utilizes younger instar larvae as test subjects, which generally exhibit higher sensitivity than older instars, facilitating the rapid evaluation of the true virulence of test samples. Using this system, we screened multiple Cry8 family proteins and discovered, for the first time, that Cry8Ca and Cry8Ga proteins exhibit high insecticidal activity against *P. striolata* larvae, with LC_50_ values of 2.243 mg/mL and 1.649 mg/mL, respectively. These findings provide novel and highly effective protein resources beyond Cry3A for the management of this challenging pest. Notably, G033A achieved ~100% mortality against *P. striolata* larvae (Figure 4), whereas purified Cry8Ca and Cry8Ga proteins achieved only ~50% mortality (Figure 6). This discrepancy likely reflects two factors. First, G033A contains multiple insecticidal proteins (Cry3Aa7, Cry1Ca, and Vip3Aa) that may act synergistically, whereas Cry8 proteins were tested individually. Second, from the perspective of resistance management, Cry8Ca and Cry8Ga proteins belong to different amino acid sequence homology groups compared to Cry3A protein [19], suggesting potential differences in their binding sites with receptors on the midgut epithelial cell membrane of the pest. This implies that they may possess distinct mechanisms of action. Resistance to a particular Cry protein in pests often arises from alterations in its specific receptor-binding pathway [20]. Baxter et al. showed that genetic mapping of the ABCC2 transporter is linked to Bt Cry1Ac resistance in two lepidopteran species, revealing that the disruption of a single receptor gene can confer high-level resistance via parallel evolution [21]. However, evidence accumulated over the past two decades has revealed a more complex landscape. Resistance can involve functional redundancy among multiple receptors (e.g., ABCC2/ABCC3) [22], constitutive activation of intracellular signaling cascades, such as the MAPK pathway, which downregulates receptor expression [23], immune evasion, and tripartite interactions with the gut microbiota [22,23]. Consequently, combining Cry proteins with different mechanisms of action (e.g., Cry3A and Cry8Ca/Cry8Ga) into composite formulations through genetic engineering or rotating their use in practical pest management could effectively delay or overcome the development and progression of pest resistance. Thus, the findings of this study provide potential tools for pest resistance management.

In summary, this study established insect-rearing techniques and an insecticidal activity assay system and discovered novel highly active Cry proteins. Collectively, these contributions provide robust support for the green control of *Phyllotreta striolata* and the development of novel Bt insecticides from both technological and resource perspectives. Future studies will build upon these findings to further elucidate the mechanisms of action of Cry8Ca and Cry8Ga proteins and explore their synergistic effects with other proteins, thereby facilitating their practical application.

## 4. Conclusions

This study established a standardized and reproducible laboratory bioassay system for *Phyllotreta striolata* larvae, maintaining natural mortality below 5% within 48 h. Using this robust platform, we discovered for the first time that Cry8Ca and Cry8Ga proteins exhibit high insecticidal activity against *P. striolata* larvae, with LC50 values of 2.243 mg/mL and 1.649 mg/mL, respectively. These novel proteins, which are phylogenetically distinct from the currently used Cry3A, offer promising alternatives for delaying pest resistance. This study provides a methodological foundation for evaluating biocontrol agents and identifies new candidate proteins for developing next-generation Bt insecticides against this globally significant pest.

## 5. Materials and Methods

### 5.1. Experimental Insects and Bacterial Strains

The *Phyllotreta striolata* individuals used for the bioassays in this study were obtained from a laboratory-reared population maintained at the Langfang Scientific Research Base of the Chinese Academy of Agricultural Sciences [24]. The *Bacillus thuringiensis* (Bt) strains used in this study are listed in Table 1. Bt strains were cultured in 1/2 liquid LB medium at 30 °C with shaking at 220 rpm.

### 5.2. Screening of Optimal Host Plants for Phyllotreta striolata in the Field

In September, five crop species—*Brassica rapa* var. *pekinensis*, *Brassica rapa* var. *chinensis*, *Raphanus sativus* var. *radicula*, *Brassica juncea* var. *foliosa* and *Brassica juncea* var. *megarrhiza*—were planted in five separate mesh house enclosures, each measuring 4 m × 12 m. Only one crop species was cultivated in each enclosure. Eleven rows of crops were sown with a spacing of 3 cm between rows. After crop germination, 1000 *P. striolata* adults (male:female = 1:1) were released into each mesh house. Yellow sticky trap assays were conducted on the 25th day after sowing. Using an equidistant sampling method, each 4 m × 12 m enclosure was divided into three 4 m × 4 m plots. A bamboo pole was inserted at the center of each plot, and two yellow sticky traps (25 cm × 40 cm) were attached back-to-back to the pole at a height of 20 cm above the ground. Given that adult *P. striolata* only emerge from the soil or hide in dark crevices to feed on host plant surfaces during daylight hours, and burrow into the soil or hide under leaves and in crevices at night [25], data were recorded only during daytime (6:00 to 18:00). The number of beetles trapped on the yellow sticky traps was counted every 2 h. The traps were replaced daily, and data were collected for three consecutive days. Feeding punctures on crop leaves were investigated 15, 25, and 35 days after sowing. Each investigation was conducted at 6 a.m. For each host plant species, 50 plants were randomly selected and divided into 10 groups of five plants each. Feeding punctures with diameters of approximately 0.2–0.5 mm were also examined. The number of penetrating holes on the leaves was counted, followed by counting the number of non-penetrating punctures on both the adaxial and abaxial leaf surfaces.

### 5.3. Cultivation of Host Plants

To compare the oviposition amount of *Phyllotreta striolata* on plants grown under different cultivation methods, *Brassica rapa* var. *chinensis*, *Brassica rapa* var. *pekinensis*, and *Brassica juncea* var. *foliosa* were cultivated using both hydroponic and soil-based methods in a greenhouse.

Hydroponic cultivation: One hundred seeds with plump, round morphologies and similar sizes were selected and immersed in water at 50 °C for 10 s. The seeds were then placed into 15 cm × 15 cm medical gauze, moistened, and the gauze was folded twice before being placed in a disposable cup. The cups were covered with black cotton cloth and placed in an incubator at 28 °C for germination induction. After three days, well-developed seedlings were carefully removed from the gauze using forceps. The roots were inserted downward into planting sponges that were fully saturated with water. The sponges were placed on the grid plate of a seedling tray, and 30 L of Hoagland nutrient solution [26] was added to the bottom. When the seedlings had grown to the 3–4 true leaf stage, they were transferred to 28 °C with sponges into planting baskets, which were then placed on a hydroponic cultivation rack. The hydroponic rack was placed inside a 125 cm × 75 cm × 120 cm insect rearing cage (80 mesh) and maintained in a greenhouse.

Soil-based cultivation: Black square pots (height: 9.5 cm, top diameter: 10.3 cm, bottom diameter: 8.5 cm) were filled with a 1:1 mixture of vermiculite and peat. Germinated seeds (with emerging radicles) were placed at the center of each pot. The soil was thoroughly watered, and the pots were transferred to a greenhouse for continued cultivation.

### 5.4. Quantification of Adult Phyllotreta striolata Oviposition

For oviposition assays, 100 adult *P. striolata* individuals with a male-to-female ratio of 1:3 were placed in insect rearing cages (50 cm × 50 cm × 50 cm, 80 mesh).

Temperature gradient experiment: Mature roots of soil-cultured *Brassica rapa* var. *pekinensis* with similar growth status was trimmed to a length of 15 cm and wrapped together with black flannel cut into 15 cm × 15 cm pieces; the assembled root-cloth units were moistened. Each wrapped root was placed in a disposable cup, which was positioned at the center of a rearing cage. The cages were placed in incubators set to different temperatures (22, 24, 26, 28, 30, and 32 °C) under constant conditions of 12,000 Lux light intensity, a 14:10 h (light:dark) photoperiod, and 40% relative humidity. After three days of cultivation, the cloth wraps were removed from the pots. The *Brassica rapa* var. *pekinensis* were rinsed with water, and the rinse water was filtered through a black cotton cloth. The number of eggs deposited on both the filtering cloth and various oviposition substrates was counted. Each temperature treatment was performed in triplicates.

Substrate and host plant combination experiment: Given that effective oviposition methods often utilize combinations of different host plants and substrates to meet the oviposition requirements of *P. striolata*, we compared the number of eggs oviposited under different substrate and host plant combinations using a method similar to that used in the temperature gradient experiment described above. In the rearing cages, 15 cm-long roots of soil-cultured *B. rapa* var. *pekinensis*, *B. rapa* var. *chinensis* and *B. juncea* var. *foliosa* were each wrapped and moistened with 15 cm × 15 cm pieces of four different substrates: black flannel, black cotton cloth, black linen cloth, and black nonwoven fabric. Black cloth was selected as the oviposition substrate because it provides a dark, light-shielded environment that mimics the natural soil conditions where *P. striolata* adults oviposit and larvae develop. In the field, females lay eggs in dark, moist soil cracks near host plant roots, and the newly hatched larvae remain in the dark soil environment [27]. Thus, the use of a black substrate under laboratory conditions simulates this natural dark habitat, encouraging normal oviposition behavior and maintaining the viability of the larvae. Each wrapped root was placed in a disposable cup, and the cups were positioned at the four corners of a rearing cage. The cages were placed in incubators set to 28 °C, with a light intensity of 12,000 lx, a 14:10 h photoperiod, and 40% relative humidity. After three days, the cloth wraps were removed. The roots were rinsed with water, and the rinse water was filtered through a black cotton cloth. The number of eggs deposited on the filtering cloth and each substrate was counted. Each treatment combination was performed in triplicates.

### 5.5. Larval Bioassay for Phyllotreta striolata

Agar solution (20 mg/mL) was prepared, sterilized by autoclaving, and poured into Petri dishes (6 cm diameter) to solidify for later use. Fresh, plump roots of *Brassica juncea* var. *megarrhiza* were transversely sliced into thin sections approximately 1 mm thick using a vegetable grater. The root slices were immersed for 5 min in different concentrations of bacterial suspensions containing 0.15% Tween-20, removed, air-dried, and placed onto agar-containing Petri dishes. Active second-instar *P. striolata* larvae were selected from the oviposition cloth. Using an insect pin, 24 or 30 larvae were gently transferred to each root slice. The Petri dishes were placed in an incubator at 28 °C and maintained in the dark. Larval mortality was recorded 48 h post-treatment. The negative control group was treated with root slices immersed in sterile water or 10 mmol/L PBS (pH 7.5). Each treatment was performed in triplicate. Mortality rate (%) was calculated as follows: (number of dead larvae/total number of larvae tested) × 100. The corrected mortality rate (%) was calculated using Abbott’s formula: (treatment mortality − control mortality)/(1 − control mortality).

### 5.6. Extraction and Quantification of Cry Proteins

The extraction method was performed as described by Zhou et al. [28] with minor modifications to the protocol. Briefly, Bt strains were cultured in 1/2 liquid LB medium until 50% of the spores were lysed. The culture was then centrifuged at 8000 rpm for 10 min at 4 °C to collect the spore-crystal mixture. The pellet was washed twice with 1 M NaCl. The washed pellet was resuspended in 50 mM Na_2_CO_3_ extraction buffer (30 mL buffer per liter of the original culture) supplemented with 10 mM dithiothreitol (DTT). The suspension was gently shaken at 70 rpm for 1 h at low temperature. After centrifugation at 8000 rpm for 10 min at 4 °C, 1/7 volume of 4 M NaAc buffer was slowly added to the supernatant, and the mixture was incubated at low temperature for 4 h. Following another centrifugation at low temperature, the pellet was washed twice with sterile water and centrifuged again. The final pellet was dissolved in 0.01 M PBS to obtain Cry8 protoxin proteins. The extracted proteins were examined using 10% SDS-PAGE. Protein bands were quantified using the ImageJ software.

### 5.7. Statistical Analysis

Data were collated using Microsoft Excel software. Statistical analyses and graphical visualizations were performed using GraphPad Prism 10 software. Data are expressed as means ± standard error of the mean (SE) and checked for normal (Gaussian) distribution using the Shapiro–Wilk test. Comparisons between the two groups were analyzed using independent samples t-tests. Comparisons among multiple groups were analyzed using one-way analysis of variance (ANOVA), followed by Duncan’s new multiple range test for multiple comparisons.

## Figures and Tables

**Table 1:** Strains were used in this study.

Strains	Characteristics	Source
G033A	The genetically engineered strain, which contained *cry1Aa*, *cry1Ac*, *cry1Ca*, *cry2Ab*, and *cry3Aa7* genes.	[7]
HD3Aa	HD73^-^ strain containing plasmid pSTK-*cry3Aa7*, Km ^r^	This lab
HD8Ca	HD73^-^ strain containing plasmid pSTK-*cry8Ca2*, Km ^r^	This lab
HD8Ea	HD73^-^ strain containing plasmid pSTK-*cry8Ea1*, Km ^r^	This lab
HD8Fa	HD73^-^ strain containing plasmid pSTK-*cry8Fa1*, Km ^r^	This lab
HD8Ga	HD73^-^ strain containing plasmid pSTK-*cry8Ga1*, Km ^r^	This lab

Km ^r^: indicates resistance to kanamycin.

## Data Availability

The original contributions presented in this study are included in the article. Further inquiries can be directed to the corresponding authors.
